# *TULP3* Regulates Proliferation and Differentiation of 3T3-L1 Preadipocytes Through the Hedgehog Signaling Pathway

**DOI:** 10.3390/biology14040369

**Published:** 2025-04-03

**Authors:** Xinlin Jin, Yu Zhang, Yunzhou Wang, Hongzhen Cao, Qi Song, Jingsen Huang, Wei Chen, Hui Tang, Yongqing Zeng

**Affiliations:** 1 Shandong Provincial Key Laboratory for Livestock Germplasm Innovation & Utilization, College of Animal Science and Technology, Shandong Agricultural University, Tai’an 271018, China; xinlin_jin@163.com (X.J.); zhangyu97127@163.com (Y.Z.); hongzhencao@163.com (H.C.); qisong114@163.com (Q.S.); hjs990606@163.com (J.H.); wchen@sdau.edu.cn (W.C.); tanghui@sdau.edu.cn (H.T.); 2 Department of Veterinary Medicine, Shandong Vocational Animal Science and Veterinary College, Weifang 261061, China; wyzwin01@163.com

**Keywords:** *TULP3*, adipose, precursor adipocytes, Hedgehog signaling pathway

## Abstract

The TULP family was first identified in progressively obese mice, and *TULP3*, as a member of its family, has been much studied in tumor cells, but studies on its role in adipocytes have not yet been reported. This study found that the expression of *TULP3* showed an increasing trend in the differentiation of 3T3-L1 cells, and overexpression of *TULP3* enhanced the proliferation and differentiation capacity of the cells, while inhibition caused the opposite result. *TULP3* is a negative regulator of the Hedgehog signaling pathway, which can control lipid metabolism in adipose tissues, but whether *TULP3* can play a role in adipose tissues through the Hedgehog signaling pathway is not yet known. It was experimentally found that *TULP3* could promote adipogenic differentiation of precursor adipocytes by inhibiting the activity of the Hedgehog signaling pathway. Our results elucidate the role of TULP3 in the generation of precursor adipocytes and provide useful information for a deeper understanding of the molecular mechanisms of adipocytogenesis, which will contribute to the improvement of the treatment of adipose tissue dysfunction or uncontrolled adipogenesis-related diseases.

## Simple Summary

Adipose tissue is a metabolically active organ that plays a crucial role in maintaining overall health and in the pathogenesis of various diseases. *TULP3* is prominently expressed in adipocytes, yet its specific function within adipose tissue remains unclear. In this study, we observed that *TULP3* expression increased during the differentiation of 3T3-L1 preadipocytes and found that it promoted both the proliferation and differentiation of these cells. Moreover, *TULP3* acts as a negative regulator of the Hedgehog signaling pathway, inhibiting its activity to further support the differentiation of precursor adipocytes. These findings uncover the role of *TULP3* in adipocyte differentiation and suggest that targeting *TULP3* may offer a promising strategy for treating obesity and related metabolic disorders.

## 1. Introduction

Adipose tissue is the largest, metabolically active energy store in the animal organism, and its formation is a complex process. The fat content of an animal body depends largely on the number and size of adipocytes, which means that the process of proliferation, differentiation, and growth and hypertrophy of precursor adipocytes determines the process of adipose formation [1]. The formation of adipocytes is also more complex. The formation of mature adipocytes is generally divided into two phases, which are the directional differentiation of MSCs to precursor adipocytes and the differentiation of precursor adipocytes to mature adipocytes [2,3], known as terminal differentiation. The study of the proliferation and differentiation of precursor adipocytes can reflect the process of adipogenesis and provide a theoretical basis for the pathogenesis of adipose-related diseases as well as for the improvement of meat quality in livestock and poultry.

*TULP3* is a member of the Tubby gene family [4], which was first identified in the mouse genome and has since been followed by the discovery of several proteins with Tubby structural domains [5], hence the name Tubby-like proteins. *TUB* was the first member of the Tubby gene family to be identified in obese mice [6], and splicing defects in the carboxyl-terminal intron of *TUB* lead to the maturity-associated obesity and delayed obesity associated with insulin resistance [7]. *TULP3* is the family member most closely related to TUB, and it is highly expressed in adipose tissue [8] and is known to promote cancer development, embryonic development, and neurodevelopment [9]. Therefore, we speculated that *TULP3* would have some effect on adiposity, and through the previous work in our laboratory, we found that *TULP3* did differ within the high and low intramuscular adipose groups [10].

Nowadays, most of the studies on *TULP3* focus on its effects on tumors and embryonic development in mice, including the fact that *TULP3* is highly expressed in patients with abdominal aortic aneurysm, pancreatic cancer, and colorectal cancer, and that it has been shown to promote cancer progression [11,12,13]. Disruption of the *TULP3* gene affects the development of neuronal cell populations [14]. *TULP3* is a key deterrent of the Hedgehog signaling pathway, which controls lipid metabolism in adipose tissue [15]. Matz-Soja et al. [16] found that conditional knockout of the Smo gene in adult mice down-regulated the activity of the Hedgehog signaling pathway and could induce hepatic steatosis. Breitrück et al. [17] found that the Hedgehog signaling pathway also maintains hepatic lipid metabolism homeostasis by balancing circadian patterns. In addition, the exacerbation of steatosis in patients with prosencephalic anencephaly caused by mutations in the Hedgehog signaling pathway demonstrated that the Hedgehog signaling pathway has an anti-hepatic steatosis effect in humans [18]. Therefore, we explored the role of *TULP3* in terms of whether it influences the prerequisite adipocytes through the Hedgehog signaling pathway.

In this study, we show that *TULP3* promotes the proliferation and differentiation of precursor adipocytes by inhibiting the Hedgehog signaling pathway. The results of our study demonstrated the positive role of *TULP3* in primary preadipocytes, which would contribute to the improvement of body fat quality in livestock and poultry for the treatment of adipose tissue dysfunction-related diseases.

## 2. Methods and Materials

### 2.1. Cell Culture and Differentiation

3T3-L1 precursor adipocytes were cultured in high glucose Dulbecco’s modified Eagle medium (DMEM, Gibco, New York, NY, USA) supplemented with 10% fetal bovine serum (FBS, Gibco, New York, NY, USA) and 0.5% penicillin/streptomycin (Solarbio, Beijing, China), at 37 °C in humid air containing 5% CO_2_.

After the 3T3-L1 precursor, adipocytes grew to 100% confluence and growth inhibition occurred; the culture was continued for 48 h to allow the cells to exit the growth cycle. Then, the induced differentiation medium A solution was replaced to start inducing differentiated cells, which was recorded as day 0 (D0). The cells were incubated at 37 °C, saturated humidity, in a 5% CO_2_ incubator for 2 days. After two days, it was replaced with Induced Differentiation Medium B solution to continue incubation for 2 days. On day 4 (D4), the medium was replaced with normal medium, and the medium was changed every two days until differentiation into mature adipocytes. Sampling was performed at different periods, and the start of induction was recorded as D0. Cells on days D0, D2, D4, D6, D8 and D10 of differentiation were collected for subsequent experiments. Induced differentiation medium A: 10% fetal bovine serum DMEM + 0.5 mmol/L IBMX (Solarbio, Beijing, China) + 1 μmol/L DEX (Solarbio, Beijing, China) + 2 μmol/L insulin (Solarbio, Beijing, China). Differentiation medium B: 10% fetal bovine serum DMEM + 2 μmol/L insulin.

### 2.2. Cell Transfection

Transfection of expression vectors into 3T3-L1 precursor adipocytes was performed using Lipofectamine 2000 (Thermo Fisher Scientific, Carlsbad, CA, USA). And the cells were inoculated in six-well cell culture plates prior to transfection. Transfection was carried out when the cells were 60% to 80% grown. Preparation of siRNA transfection complex (dosage per well), liquid A: 100 uL Opti-MEM (Gibco, New York, NY, USA) + 5 uL siRNA; liquid B: 100 uL Opti-MEM + 5 uL Lipofectamin2000 reagent. Preparation of *TULP3*-pcDNA3.1 transfection complex, liquid A: 100 uL Opti-MEM + 5 ug overexpression plasmid; liquid B: 100 uL Opti-MEM + 10 uL Lipofectamin2000 reagent. Afterwards, the culture plates were kept at room temperature for 5 min, then the B liquid was added to the A liquid and left to rest for 20 min. Each well of cells was given 1 mL of preheated Opti-MEM. A total of 200 uL of the mixture that was already prepared was added to the cells of each well and gently mixed in. The complete medium was replaced after 4–6 h of incubation.

### 2.3. Plasmid Construction

The following primer sequences for *TULP3* cDNA were synthesized as follows (Table 1):

The full-length cDNA of *TULP3* was obtained by PCR, and the PCR product was recovered by gel and sequenced to ensure the correct sequence. The PCR product and plasmid were digested with Hind III and Xho I (Takara, Shiga, Japan), and then the *TULP3* fragment was ligated into pcDNA-3.1 vector with T4 DNA ligase (Takara, Shiga, Japan) to generate pcDNA3.1-*TULP3*, which was sequenced to ensure sequence correctness and was transient plasmid-based. An amount of 10 uL of the recombinant plasmid was added to 50 μL of DH5α receptor cells, transformed, and the plasmid amplified in bacterial cells.

### 2.4. Oil Red O Staining

The cells were raised to the required number of days, the cell culture medium was discarded and washed with PBS 3 times; the cells were fixed with 4% paraformaldehyde for 40 min, and the solution was discarded and washed with PBS 3 times. Oil Red O staining solution was then added to cover the cells and stained for 20 min at room temperature; then, the staining solution was discarded, and the cells were washed with 60% isopropyl alcohol to remove the residual Oil Red O staining solution for observation.

### 2.5. CCK-8 Cell Proliferation Assay

The CCK-8 kit method was used to calculate the proliferative activity of the cells. The cell suspension was added to 96-well plates in the amount of 100 μL/well, with 6 replicates in each group, and an equal amount of culture medium was added to 6 empty wells as a blank control. After 24 h of cell culture, the cells were transfected with overexpression plasmid and interference fragments. After continued incubation for 24 h, 10 μL of CCK-8 solution was added to each well, and the absorbance value at 450 nm was measured by an enzyme marker (BioTek, Winooski, VT, USA) after incubation for 1~2 h in an incubator protected from light.

### 2.6. Total RNA Extraction, cDNA Synthesis, and Real-Time RT-PCR

RNA extraction kits as well as reverse transcription kits and the SYBR^®^ Green Pro Taq HS Pre-mixed qPCR kits were from Accurate Biotechnology (AG, Changsha, Hunan, China). RNA extraction and reverse transcription were performed on ice to preserve RNA integrity. Briefly, 1 µg of total RNA in a 20 µL reaction mixture reverse transcriptase was used to synthesize 1st strand cDNA using the following schedule: 37 °C for 15 min, 85 °C for 5 s, preservation at 4 °C. Gene expressions were analyzed using cDNA (2 µL) and gene-specific primers (0.2 uM, 0.4 µL) and SYBR Premix Ex Taq(2x)(10 µL). All reactions were performed in triplicate, and relative amounts of gene expressions normalized vs. controls were calculated using 2^−∆Ct^, where ∆Ct = Ct gene − Ct control. β-actin was used as an internal control. The primers used are detailed in Table 2.

### 2.7. Western Blot

3T3-L1 precursor adipocytes were lysed using RIPA lysis solution containing PMSF (Beyotime, Shanghai, China). BCA protein assay kit (Beyotime, Shanghai, China) was used to quantify protein. The treated cells were cultured to the desired number of days, washed once with PBS, and then the cells were lysed with RIPA lysis solution containing PMSF and collected into 1.5 mL centrifuge tubes. Lysates were centrifuged at 12,000× *g* for 5 min at 4 °C, and the supernatant was collected. The supernatants were subjected to BCA protein analysis (Beyotime, Shanghai, China) for protein quantification. Protein samples were diluted to the same concentration with lysate and added to SDS-PAGE up-sampling buffer, then heated at 100 °C in a metal bath for 10 min and stored at −20 °C. The 20 μg protein was loaded in each well and then separated via SDS-PAGE. Subsequently, proteins were transferred to a PVDF membrane. After blocking nonspecific sites with sealing solution, the PVDF membranes were sequentially incubated with primary antibodies overnight at 4 °C, washed three times with TBST, and secondary antibodies for 1 h. ECL luminescent solution was added to the PVDF membrane and incubated for 5 min away from light, then the protein bands were detected in a chemiluminescent imager.

### 2.8. Immunofluorescence and Laser Confocal Microscopy

Cells were inoculated on 6-well plate cell crawls at a density of 1 × 10^5^ cells/mL, rinsed three times by adding pre-cooled phosphate buffer, then fixed with pre-cooled 4% paraformaldehyde for 30 min, and rinsed three times by adding phosphate buffer. The cells were permeabilized using 0.1% Triton X-100 (Solarbio, Beijing, China) for 40 min at room temperature, added phosphate buffer rinsed three times, and the cells closed in 37 °C plus 5% fetal bovine serum for 1 h. The closure solution was removed, not washed, and the primary antibody with 1:100 dilution of blocking buffer was added, and the cells were incubated overnight at 4 °C, added phosphate buffer rinsed three times. Sheep anti-rabbit FITC secondary antibody (1:100) was added, incubated at 37 °C, protected from light for 1 h. Add phosphate buffer to rinse three times, stained with 100 μL of DAPI at room temperature, incubated at 37 °C, protected from light for 5 min. Add phosphate buffer to rinse two times, remove phosphate buffer, and obtain fluorescence images by using laser confocal microscopy.

### 2.9. Activation and Inhibition of the Hedgehog Signaling Pathway

Throughout the process of starting cell culture, including induction of differentiation, 10 µmol/L of the Hedgehog signaling pathway activator Pu or inhibitor Cy was added at each change of solution, respectively, along with the same concentration of DMSO as a control.

### 2.10. Statistical Analysis

All data were analyzed using IBM SPSS Statistics 20 software, and the statistical significance of differences among groups was examined using a *t*-test or one-way ANOVA analysis. Images were plotted using GraphPad Prism 5.0, with data for each group expressed as Mean ± SEM. Statistical significance is expressed as * *p* < 0.05; ** *p* < 0.01; *** *p* < 0.001.

## 3. Results

### 3.1. Expression Pattern and Localization of TULP3 During the Differentiation

**Figure 1:**
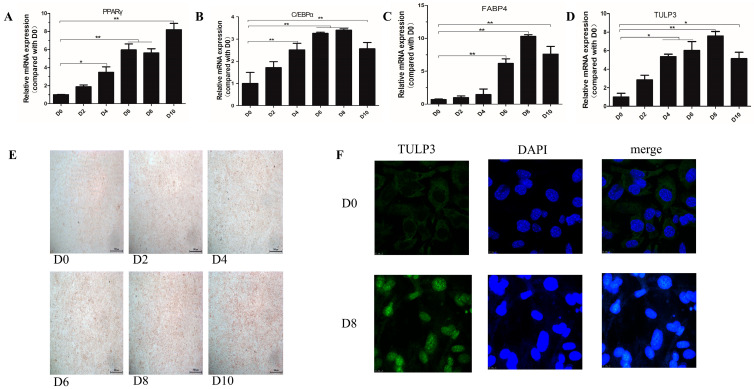
Induction of lipidogenic differentiation of 3T3-L1 cells for assay of differentiation effects. (**A**) Expression of *PPARγ* during cell differentiation. (**B**) Expression of *C/EBPα* during cell differentiation. (**C**) Expression of *FABP4* during cell differentiation. (**D**) Expression of *TULP3* during cell differentiation. (**E**) Oil Red O staining diagram of cells; scale bars represent 500 µm. (**F**) Analysis performed by InCell-2000 confocal microscope. Blue and green fluorescence indicate localization of nucleus (DAPI) and *TULP3*, respectively. Scale bars represent 10 µm. * *p* < 0.05, ** *p* < 0.01, *n* = 3.

To investigate the effect of TULP3 on lipid differentiation of 3T3-L1 cells, cells were collected at 0, 2, 4, 6, 8 and 10 d after differentiation. We examined the expression levels of TULP3 and lipid formation differentiation marker genes at different stages of 3T3-L1 cell differentiation, and found that compared with D0, the expression of *PPARγ* and *C/EBPα* started to increase significantly at D4 and was most significant at day D6. And the expression trend of *TULP3* was similar to that of lipidogenic differentiation marker genes (Figure 1A–D). Oil Red O staining of the cells during the process of cell differentiation revealed that the number of lipid droplets became dense as the cell differentiation progressed (Figure 1E). Subcellular localization of *TULP3* using laser confocal showed that *TULP3* protein was expressed outside the nucleus at D0, whereas the expression of *TULP3* protein in the nucleus was significantly higher at D8 (*p* < 0.05) (Figure 1F). These findings demonstrate that TULP3 protein undergoes translocation from the cytoplasm into the nucleus during cell differentiation. Taken together, this induction method was shown to be feasible and the precursor adipocyte lipidogenic differentiation was associated with the expression of the *TULP3* gene, which was transferred from the cytoplasm into the nucleus after being induced to differentiate.

### 3.2. TULP3 Gene Promotes Proliferation of 3T3-L1 Cells

**Figure 2:**
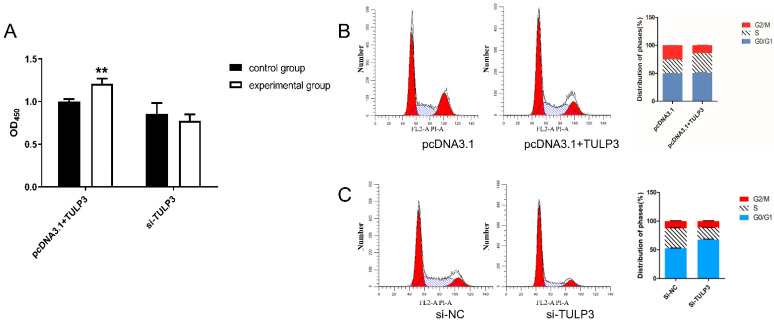
Detection of proliferative capacity of precursor adipocytes by *TULP3*. (**A**) CCK-8 detects proliferative activity of *TULP3* on precursor adipocytes. (**B**) Effect of transfection of pcDNA3.1-*TULP3* on the cell cycle of precursor adipocytes. (**C**) Effect of transfection of si-*TULP3* on the cell cycle of precursor adipocytes. ** *p* < 0.01, *n* = 3.

The interference fragments and overexpression vectors were transfected into cells, and when the cell density reached about 80%, the cell proliferation activity was detected using the CCK-8 kit. In addition, the cells were stained with PI and assayed by flow cytometry. It was found that when overexpressing *TULP3*, the cells produced more metazan and were more active, with higher absorbance values at 450 nm. While interfering with *TULP3* had a tendency to reduce the proliferative activity of the cells, the difference was not significant (Figure 2A). The results of flow cytometry showed that the proportion of S-phase cells in cells overexpressing *TULP3* (35.24% ± 0.68%) was significantly higher (*p* < 0.05) than that of control cells (24.79% ± 0.74%) (*p <* 0.05), while the proportion of G2/M-phase cells (14.30% ± 0.79%) was significantly lower than that of control (25.43% ± 0.12%) (*p* < 0.05) (Figure 2B). Si-*TULP3* group had a significantly higher proportion of G0/G1-phase cells (67.71% ± 0.98%) than that of the si-NC group (52.55% ± 0.64%) (*p* < 0.05). The proportion of S-phase cells in the si-*TULP3* group (21.21% ± 0.14%) was significantly lower than that of the si-NC group (34.83% ± 1.38%) (*p* < 0.05) (Figure 2C). The above results suggest that overexpression of *TULP3*, promotes DNA synthesis in S-phase cells, which promotes the proliferation level of the cells. And interference with TULP3 may inhibit DNA synthesis in S-phase cells, which leads to the blockage of cells in G0/G1 phase.

### 3.3. TULP3 Promotes Precursor Adipocyte Differentiation

**Figure 3:**
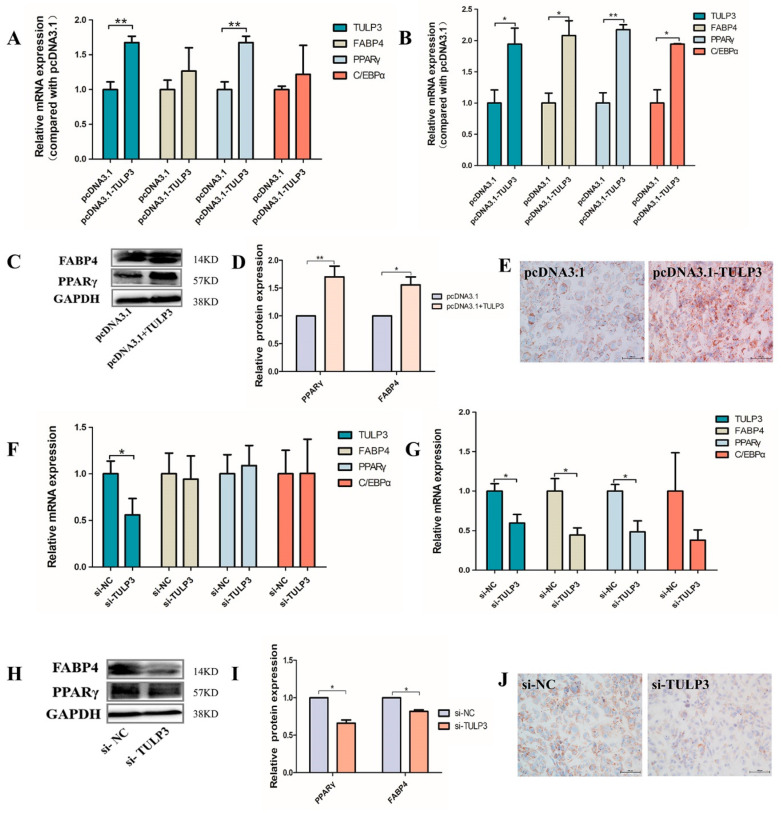
Effects of overexpression and interference with *TULP3* on preadipocyte differentiation. (**A**) Expression of *TULP3*, *PPARγ*, *C/EBPα* and *FABP4* in preadipocytes differentiated on D0. (**B**) Expression of *TULP3*, *PPARγ*, *C/EBPα* and *FABP4* in preadipocytes differentiated on D8. (**C**,**D**) The protein levels of PPARγ and FABP4 were detected by Western Blot on day D8, and the band intensity was analyzed by Image J 1.49 analysis software. (**E**) Oil Red O staining diagram of cells, Scale bars represent 100 µm. (**F**) Expression of *TULP3*, *PPARγ*, *C/EBPα* and *FABP4* in preadipocytes differentiated on D0. (**G**) Expression of *TULP3*, *PPARγ*, *C/EBPα* and *FABP4* in preadipocytes differentiated on D8. (**H**,**I**) The protein levels of PPARγ and FABP4 were detected by Western Blot on day D8, and the band intensity was analyzed by Image J analysis software. (**J**) Oil Red O staining images of cells. Scale bars represent 100 µm. * *p* < 0.05, ** *p* < 0.01, *n* = 3.

*TULP3* overexpression vector was transfected into precursor adipocytes and induced differentiation, the cells were collected at D0 and D8, and the mRNA expression levels of *PPARγ*, *FABP4*, and *C/EBPα* were detected. Cells were harvested and a cell scraper used on D8, and PPARγ and FABP4 protein expression levels were detected using Western Blot and stained with Oil Red O. At D0, the expression of *PPARγ* in the pcDNA3.1-*TULP3* group was significantly higher than that of the control group (*p* < 0.05) (Figure 3A). At D8, the expression of *PPARγ*, *FABP4* and *C/EBPα* in the pcDNA3.1-*TULP3* group was significantly higher than that of the control group (*p* < 0.05) (Figure 3B). Western Blot results and fluorescence quantification results were consistent (Figure 3C,D). For D8-cells stained with Oil Red O, the number of lipid droplets in the pcDNA3.1-*TULP3* group was significantly more than that in the control group (Figure 3E). Thus, overexpression of *TULP3* could promote the differentiation of precursor adipocytes.

In addition, we transfected the interfering fragments into cells. D0 and D8 cells were harvested with a cell scraper, and mRNA expression levels of *PPARγ*, *FABP4* and *C/EBPα* were detected by RT-qPCR. Protein expression levels of PPARγ and FABP4 were detected using staining with Oil Red O and Western Blot against differentiated day D8 cells. At D0, there was no difference between the interference group and the control group (Figure 3F). At D8, the expression of *PPARγ* and *FABP4* in the interference group was significantly lower than that in the control group (*p* < 0.05) (Figure 3G). The Western Blot results were consistent with the fluorescence quantification results (Figure 3H,I), and the number of lipid droplets in the interference group was significantly less than that in the control group (*p* < 0.05) (Figure 3J). Therefore, interfering with the expression of *TULP3* could inhibit the differentiation of precursor adipocytes.

### 3.4. TULP3 Inhibits Hedgehog Signaling Pathway

**Figure 4:**
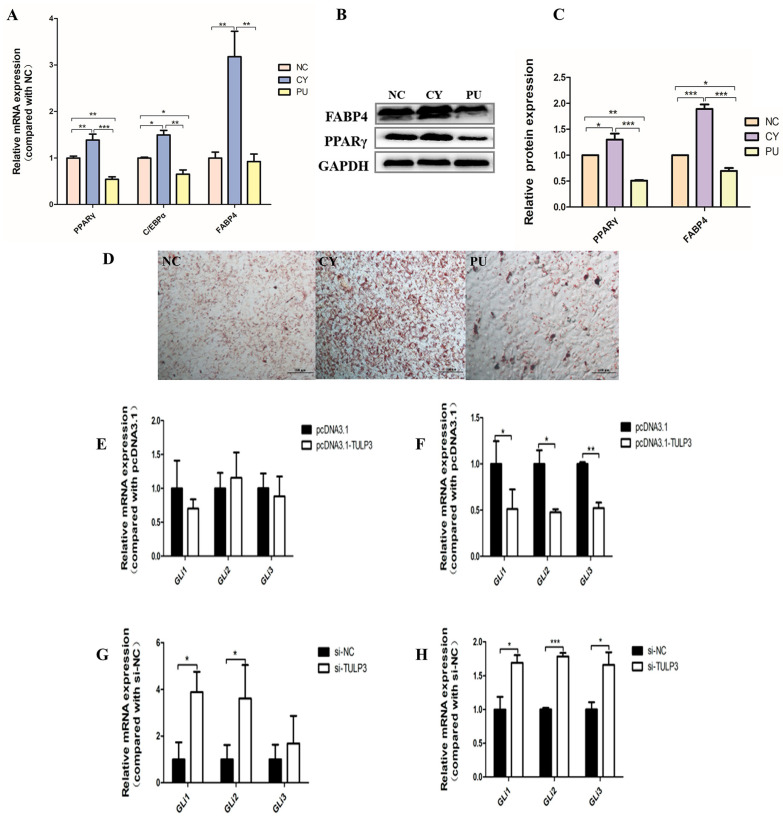
Hedgehog signaling pathway on precursor adipocyte differentiation and the effect of TULP3 on the Hedgehog signaling pathway. (**A**) Expression levels of *PPARγ*, *C/EBPα* and *FABP4* in preadipocytes differentiated on D8. (**B**,**C**) The protein levels of PPARγ and FABP4 were detected by Western Blot on day D8, and the band intensity was analyzed by Image J analysis software. (**D**) Oil Red O staining diagram of cells. Scale bars represent 100 µm. (**E**) Expression levels of *Gli1*, *Gli2* and *Gli3* in adipocytes overexpressing *TULP3* at D0 differentiation. (**F**) Expression levels of *Gli1*, *Gli2* and *Gli3* in adipocytes overexpressing *TULP3* at D8 differentiation. (**G**) Interferes with the expression levels of *Gli1*, *Gli2* and *Gli3* in adipocytes at D0 differentiation of TULP3. (**H**) Interferes with the expression levels of *Gli1*, *Gli2* and *Gli3* in adipocytes at D8 differentiation of *TULP3*. * *p* < 0.05, ** *p* < 0.01, *** *p* < 0.001, *n* = 3.

Cells were treated with the Hedgehog signaling pathway inhibitor Cyclopamin (Cy) and activator Purmorphamine (Pu), respectively. DMSO control was used for the NC group, pathway activator was added to the PU group, and pathway inhibitor was added to the CY group. When cells were cultured and induced to differentiate, 10 µmol/L of pathway activator or inhibitor were added at each fluid change. At D8, the expression of *PPARγ*, *FABP4*, and *C/EBPα* was significantly higher in the CY group than in the other groups (*p* < 0.05), while in the PU group, the expressions of *PPARγ* and *C/EBPα* were significantly lower than those in the other groups (Figure 4A). Western Blot results and fluorescence quantification results were consistent (Figure 4B,C). Compared with the control group, at D8 of precursor adipocyte-induced differentiation, the lipid droplet density was significantly increased in the CY group (*p* < 0.05) and decreased in the PU group (*p* < 0.05) (Figure 4D). Therefore, the Hedgehog signaling pathway may exert an inhibitory effect on lipidogenic differentiation during cell differentiation.

Gli expression was detected in differentiated D0 and D8 cells after overexpression or interference with *TULP3*. In D0 cells, there was no significant change in *Gli*, and on day D8, the expression of *Gli1*, *2*, and *3* in the pcDNA3.1-*TULP3* group was significantly lower than that in the control group (*p* < 0.05) (Figure 4E,F). At D0, the expression of *Gli1*, *2* and *3* in the si-*TULP3* group was significantly higher than that in the control group (*p* < 0.05). At D8, the expression of *Gli1*, *2* and *3* in the si-*TULP3* group was significantly higher than that in the control group (*p* < 0.05) (Figure 4G,H). It can be concluded that *TULP3* inhibited the Hedgehog signaling pathway during cell differentiation.

### 3.5. TULP3 Promotes Precursor Adipocyte Differentiation by Inhibiting the Hedgehog Signaling Pathway

**Figure 5:**
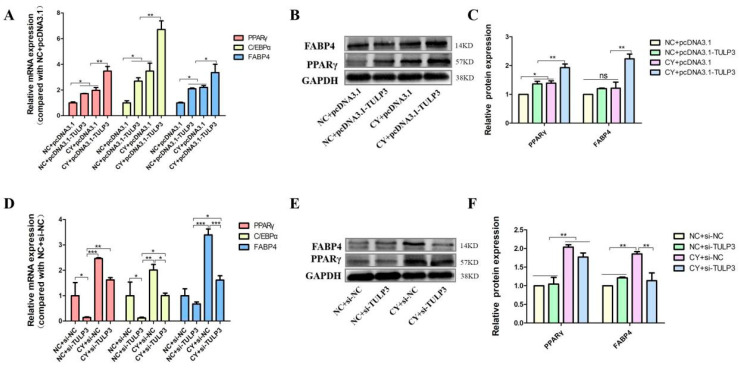
Effects of overexpression and interference with *TULP3* with simultaneous inhibition of the Hedgehog signaling pathway on precursor adipocyte differentiation. (**A**) Overexpression of *TULP3* and inhibition of the signaling pathway and expression of *PPARγ*, *C/EBPα* and *FABP4* in precursor adipocytes differentiated on D8. (**B**,**C**) The protein levels of PPARγ and FABP4 on D8 were detected by Western Blot, and the intensity of the bands was analyzed by Image J analysis software. (**D**) Interference with *TULP3* and inhibition of signaling pathways, expression of *PPAR*γ, *C/EBPα* and *FABP4* in precursor adipocytes differentiated on D8. (**E**,**F**) The protein levels of PPARγ and FABP4 on D8 were detected by Western Blot, and the intensity of bands was analyzed by Image J analysis software. * *p* < 0.05, ** *p* < 0.01, *** *p* < 0.001, ns > 0.05, *n* = 3.

To further verify that *TULP3* acts through the Hedgehog signaling pathway, the *TULP3* gene was overexpressed while cells were treated with Cy, and cells were collected on D8 of differentiation to detect the expression of marker genes for lipid differentiation. The expression of *PPARγ*, *FABP4* and *C/EBPα* in the CY + pcDNA3.1-*TULP3* group was significantly higher than that in the NC + pcDNA3.1-*TULP3* group at D8 of differentiation (*p* < 0.05). While the expression of *PPARγ*, *FABP4* and *C/EBPα* in the NC + pcDNA3.1-*TULP3* group was higher than that in the CY + pcDNA3.1 group, it was not significantly different (Figure 5A). Western Blot results and fluorescence quantification results were consistent (Figure 5B,C).

Similarly, to illustrate in reverse that *TULP3* acts through the Hedgehog signaling pathway, the interference fragment was transfected into the cells while the cells were treated with Cy, and the cells were collected to detect the expression of marker genes for lipid differentiation at D8 of differentiation. At D8, the expression of *PPARγ*, *FABP4*, and *C/EBPα* in the CY + si-*TULP3* group was significantly higher than that in the NC + si-*TULP3* group *(p* < 0.05). The expression of *PPARγ*, *FABP4*, and *C/EBPα* in the NC + si-NC group was not significantly differentiated from that of the CY + si-*TULP3* group (*p* > 0.05) (Figure 5D). The results of the Western Blot results showed that the protein expression of PPARγ in the CY + si-*TULP3* group was significantly higher than that of the NC + si-NC group (*p* < 0.05) (Figure 5E,F).

## 4. Discussion

Mutations in the *TUB* gene can lead to obesity, and *TULP3* is the family member most closely related to *TUB*, so we speculate that it may also have something to do with obesity formation. John Devane et al. observed cardiac degenerative diseases and hepatic steatosis in an adult *TULP3* knockout zebrafish model. And liver disease is usually the earliest to appear in individuals carrying a double allelic variant in the *TULP3* gene. *TULP3* gene variants cause progressive liver and kidney disease. *TULP3* interacts with DNA damage repair proteins and *SIRT1*, a key fibrosis regulator. Using mass spectrometry and immunoprecipitation experiments, it was found that *TULP3* interacts with the deacetylase *SIRT1*, which plays a role in DNA damage repair and fibrosis, and that the *TULP3* gene variant may lead to a diminished modulation of the pro-fibrotic signaling pathway by *SIRT1*. In addition, it has been reported that *TULP3* plays a regulatory role in the functioning of primary cilia [19], which play an important role in directing the expansion of white adipose tissue during obesity [20].

Adipogenesis is a two-step process: A commitment step restricting a mesenchymal precursor to the adipocyte lineage to form preadipocytes, and a differentiation step differentiating preadipocytes into insulin-sensitive mature adipocytes [21]. During the latter, preadipocytes undergo growth arrest, mitotic clonal expansion, and early and terminal differentiation [22]. We used 3T3-L1 precursor adipocytes as a research target to investigate the effect of *TULP3* on adipogenesis. Here, we confirmed that *TULP3* increased gradually with the number of days of differentiation during precursor adipocyte differentiation, and *TULP3* localization was performed on cells at D8, which was found to be translocated into the nucleus for expression. When *TULP3* was overexpressed, 3T3-L1 differentiation was positively regulated, and the expression of marker genes for adipogenic differentiation was significantly increased at both the gene and protein levels, with an increase in the number of lipid droplets. At the same time, the proliferative capacity of precursor adipocytes could be enhanced, and the proportion of s-phase cells in the cell cycle was increased. The opposite results were obtained after interfering with *TULP3*. Thus, we further demonstrated that *TULP3* promotes 3T3-L1 cell differentiation and proliferation.

Currently, there are limited reports on TULP3 and its associated pathways. Existing studies indicate that TULP3 silencing inhibits the proliferation, migration, and invasion of gastric cancer cells via the PTEN/Akt/Snail pathway [23]. Additionally, miR-4688 targeting TULP3 promotes the formation of abdominal aortic aneurysms through the STAT3/NEAT1/miR-4688/TULP3 axis [24]. Furthermore, research has shown that lithocholic acid (LCA) binds to TULP3 to activate sirtuins and the AMPK pathway, thereby delaying aging [25]. Notably, TULP3 interacts with all members of the sirtuin family through specific structural domains, and this interaction is independent of LCA presence. *TULP3* is a negative regulator of the Hedgehog signaling pathway, thereby leading to abnormalities in mouse embryo development [26,27], and it has been reported that Caudatin can down-regulate adipocyte differentiation through activation of the Hedgehog signaling pathway [28].

The Hedgehog signaling pathway not only has an important role in the growth and development of animal organisms [29] but also plays an important role in regulating adipocyte differentiation, activation of which can inhibit adipocyte differentiation and the development of white adipose tissues [30,31,32,33]. Hedgehog signaling is anti-lipogenic and osteogenic [34,35], and the Hedgehog pathway controls lipid metabolism in adipose tissues. The Hedgehog signaling pathway can inhibit the conversion of glucose to lipids and also inhibit adipocyte differentiation by inducing the expression of Wnt [29]. Downregulation of the Hedgehog signaling pathway can induce hepatic steatosis. The Hedgehog signaling pathway also maintains the homeostasis of hepatic lipid metabolism by balancing the circadian patterns.

In order to explore the mechanism by which *TULP3* promotes the differentiation of precursor adipose, we learned that *TULP3* plays a regulatory role in the Hedgehog signaling pathway. We examined the effect of Hedgehog pathway on 3T3-L1 differentiation ability by adding activators and inhibitors and found that the Hedgehog pathway was inhibitory to 3T3-L1 differentiation ability, and the expression of lipogenic marker genes in the CY group was significantly higher than that in the other groups (*p* < 0.05), while the density of lipid droplets increased. In addition, we overexpressed and knocked down TULP3. By detecting the expression of the key gene of the pathway Gli, we found that the two showed a negative correlation and overexpression of TULP3. The expression of Gli1, 2 and 3 in the pcDNA3.1-TULP3 group was significantly lower than that in the control group (*p* < 0.05), and the knockdown effect was the opposite.

To verify whether *TULP3* was functioning through the Hedgehog signaling pathway, we overexpressed *TULP3* with the addition of a Hedgehog signaling pathway inhibitor. The expression of *PPARγ*, *FABP4*, and *C/EBPα* in the CY + pcDNA3.1-*TULP3* group was significantly higher than that in the NC + pcDNA3.1-*TULP3* group (*p* < 0.05), while the expression of *PPARγ*, *FABP4* and *C/EBPα* in the NC + pcDNA3.1-*TULP3* group was not significantly different from that in the CY + pcDNA3.1 group. This suggests that overexpression of the *TULP3* gene has a similar effect with inhibition of the Hedgehog signaling pathway. Knockdown of *TULP3*, along with the addition of inhibitors, revealed that the expression of *PPARγ*, *FABP4*, and *C/EBPα* in the CY + si-*TULP3* group was significantly higher than that in the NC + si-*TULP3* group (*p* < 0.05). When *TULP3* was overexpressed, the Hedgehog signaling pathway was inhibited, and the expression of lipogenic differentiation marker genes was increased. When *TULP3* was interfered with, the activity of the Hedgehog signaling pathway was increased, and the expression of lipogenic differentiation marker genes was decreased, which confirms that *TULP3* promotes the differentiation of precursor adipocytes by inhibiting the Hedgehog signaling pathway. The operational flowchart of this experiment is included in the Supplementary Materials. TULP3 is a protein closely related to cilia function, and primary cilia are biomarkers of adipose precursor cells. TULP3 may act as an adaptor protein involved in the formation of intraciliary signaling complexes. The activity of Gli, a downstream effector molecule of the Hedgehog pathway, directly affects the expression of genes related to adipogenesis. TULP3 may affect the stability or activity of Gli by regulating its phosphorylation or ubiquitylation process or activity. TULP3 may indirectly regulate the activity of the Wnt signaling pathway by affecting the Hedgehog signaling pathway, thus affecting the proliferation and differentiation of adipocytes, which will be explored in the next step of our project.

However, the potential mechanisms involved in the role of TULP3 in adipogenesis through the Hedgehog pathway are still unclear. TULP3 is a protein closely related to cilia function, and primary cilia are biomarkers of adipose precursor cells. TULP3 may act as an adaptor protein involved in the formation of intraciliary signaling complexes, which in turn affects adipogenesis and development. Alternatively, the activity of Gli, a downstream effector of the Hedgehog pathway, directly affects the expression of adipogenesis-related genes, and TULP3 may regulate the phosphorylation or ubiquitination of Gli to influence its stability or activity. Alternatively, in synergy with other pathways, the Wnt and Hedgehog signaling pathways are cross-regulated in adipogenesis and development, and TULP3 may indirectly regulate the activity of the Wnt signaling pathway by affecting the Hedgehog signaling pathway, thereby affecting adipocyte proliferation and differentiation, which will be explored in the next step of the project.

## 5. Conclusions

In this study, we verified for the first time the promotional effect of *TULP3* on the differentiation and proliferative capacity of 3T3-L1. *TULP3* promotes lipogenic differentiation of precursor adipocytes by inhibiting the activity of the Hedgehog signaling pathway. The results of this study are useful for revealing the role of *TULP3* in influencing lipid differentiation and provide a molecular basis for the treatment of obesity diseases, as well as the improvement of livestock and poultry meat quality.

## Figures and Tables

**Table 1:** The primer sequences of PCR.

Primer	Sequence
*TULP3*-Forward (5′-3′)	GCTGGCTAGCGTTTAAACTTAAGCTTTCTCGGGGTCTGGACGCTGAT
*TULP3*-Reverse (5′-3′)	GTTTAAACGGGCCCTCTAGACTCGAGTCATTCACACGCCAGCTTGCTGTC

**Table 2:** The primer sequences of RT-qPCR.

Gene	Forward (5′-3′)	Reverse (5′-3′)
Mus-*TULP3*	TGACAAGGAGGAAGATGAGGGGGGA	GGTGTTGATAGTAGGTGGGGAAGAG
Mus-*PPARγ*	GGAAGACCACTCGCATTCCTT	GTAATCAGCAACCATTGGGTCA
Mus-*C/EBPα*	GCGGGAACGCAACAACATC	GTCACTGGTCAACTCCAGCAC
Mus-*FABP4*	AAACACCGAGATTTCCTTCA	TAACACATTCCACCACCAGC
Mus-*Gli1*	CCAAGCCAACTTTATGTCAGGG	AGCCCGCTTCTTTGTTAATTTGA
Mus-*Gli2*	ACCCCTGATCCAGCCTTCA	GTTGGCATCATTTAGACAGTTGC
Mus-*Gli3*	TGAGGGCCGTTACCATTATGA	GTCGGGCTACTAGATAAGGCA
Mus-*β-actin*	GTGACGTTGACATCCGTAAAGA	GCCGAACTCATCGTACTCC

## Data Availability

Data are contained within the article.

## Supplementary Materials

The following supporting information can be downloaded at: https://www.mdpi.com/article/10.3390/biology14040369/s1.
